# Induction of WNT11 by hypoxia and hypoxia-inducible factor-1α regulates cell proliferation, migration and invasion

**DOI:** 10.1038/srep21520

**Published:** 2016-02-10

**Authors:** Hiroyuki Mori, Yao Yao, Brian S. Learman, Kazuhiko Kurozumi, Joji Ishida, Sadeesh K. Ramakrishnan, Katherine A. Overmyer, Xiang Xue, William P. Cawthorn, Michael A. Reid, Matthew Taylor, Xiaomin Ning, Yatrik M. Shah, Ormond A. MacDougald

**Affiliations:** 1 Department of Molecular & Integrative Physiology, University of Michigan, Ann Arbor, Michigan, USA; 2 Department of Neurological Surgery, Okayama University Graduate School of Medicine, Dentistry and Pharmaceutical Sciences, Okayama, Japan; 3 Department of Internal Medicine, University of Michigan, Ann Arbor, Michigan, USA; 4 BHF/University Centre for Cardiovascular Science, The Queen’s Medical Research Institute, University of Edinburgh, Edinburgh, UK; 5 College of Animal Science and Technology, Northwest Agriculture and Forestry University, Yangling, Shaanxi, PRC

## Abstract

Changes in cellular oxygen tension play important roles in physiological processes including development and pathological processes such as tumor promotion. The cellular adaptations to sustained hypoxia are mediated by hypoxia-inducible factors (HIFs) to regulate downstream target gene expression. With hypoxia, the stabilized HIF-α and aryl hydrocarbon receptor nuclear translocator (ARNT, also known as HIF-β) heterodimer bind to hypoxia response elements (HREs) and regulate expression of target genes. Here, we report that WNT11 is induced by hypoxia in many cell types, and that transcription of WNT11 is regulated primarily by HIF-1α. We observed induced WNT11 expression in the hypoxic area of allograft tumors. In addition, in mice bearing orthotopic malignant gliomas, inhibition with bevacizumab of vascular endothelial growth factor, which is an important stimulus for angiogenesis, increased nuclear HIF-1α and HIF-2α, and expression of WNT11. Gain- and loss-of-function approaches revealed that WNT11 stimulates proliferation, migration and invasion of cancer-derived cells, and increases activity of matrix metalloproteinase (MMP)-2 and 9. Since tumor hypoxia has been proposed to increase tumor aggressiveness, these data suggest WNT11 as a possible target for cancer therapies, especially for tumors treated with antiangiogenic therapy.

Increasing evidence suggests that changes in oxygen tension play important roles in physiological processes including development, and pathological processes such as tumor promotion^1^,^2^,^3^,^4^. Cellular adaptations to sustained hypoxia are in part mediated by hypoxia-inducible factor (HIF). HIF is a heterodimeric transcription factor consisting of an oxygen-sensitive alpha subunit (HIF-1α or HIF-2α) and a constitutively expressed beta subunit, aryl hydrocarbon receptor nuclear translocator (ARNT, also known as HIF-1β)^2^,^3^,^5^,^6^. In normoxia, HIF-1α and HIF-2α are rapidly hydroxylated, and degraded by the ubiquitin-proteasome pathway^2^,^3^,^5^,^6^. In hypoxia, stabilized HIF-1α and HIF-2α bind to ARNT to regulate downstream target gene expression^1^,^2^,^5^,^6^. HIF-1α and HIF-2α regulate distinct and partially overlapping sets of target genes^2^,^4^,^6^. Since ARNT is not responsive to oxygen and is present in excess, HIF-1α and HIF-2α protein levels determine HIF transcriptional activity. Many genes are directly or indirectly regulated by HIFs, and HIF-mediated pathways are essential cellular responses to hypoxia such as metabolic adaptation, angiogenesis, erythropoiesis, and cell growth and differentiation^1^,^2^,^3^,^4^,^6^,^7^. One well-established indirect mechanism for HIF-α to influence downstream cellular events is through regulation of WNT signaling proteins such as β-catenin^8^,^9^.

WNT family members are highly-conserved secreted proteins with post-translational modifications such as glycosylation and palmitoylation^10^,^11^. Although WNTs were originally classified as signaling through canonical (via β-catenin) or non-canonical pathways, recent studies indicate that many WNTs activate numerous pathways, depending on the expression profile of WNT receptors (including Frizzled family members), LRP5, LRP6, ROR1, ROR2 and RYK^10^,^11^,^12^. One of the WNTs considered to work almost exclusively through non-canonical signaling mechanisms is WNT11, which signals through Frizzled 4, 5, and 7 to activate CaMKII, PKC, and RhoA^12^. Expression of WNT11 is regulated by myriad factors including Ret/GDNF signaling, estrogen/estrogen-related receptor α (ERRα), β-catenin and TCF/LEFs^12^, and is found extensively throughout embryonic^13^,^14^ and adult tissues^15^. Crucial roles for WNT11 during embryogenesis and organogenesis have been elucidated using genetically engineered animals^16^,^17^,^18^, and effects on specification, development, and cardiomyocyte maturation in the heart are well described^12^,^13^,^17^,^19^. Moreover, WNT11 is highly expressed in several cancers and cancer-derived cell lines, where it is implicated in proliferation, survival of progenitor-like cells, and migration and/or invasion^12^,^13^,^15^,^20^,^21^,^22^.

In this manuscript, we further investigated the mechanistic link between WNT11 and cancer. We report that WNT11 is induced by hypoxia in many cell types, and that transcription of WNT11 is regulated primarily by HIF-1α. Elevated endogenous WNT11 increases activity of MMP2 and MMP9, and promotes proliferation, migration and invasion of cancer-derived cells. Finally, elevated WNT11 expression was observed in the hypoxic area of allograft tumors and in human malignant glioma xenografts after treatment with antiangiogenic therapy. Although antiangiogenic therapy is thought to hold significant potential for the treatment of cancer, limited effectiveness, and increased tumor invasiveness and metastasis have been reported^23^,^24^,^25^. Taken together, our results provide a possible mechanism by which WNT11 induced by hypoxia and the HIF pathway regulates cell migration and invasion through activation of MMPs, and this could be a potential mechanism that drives the deleterious action of antiangiogenic therapy.

## Results

### Hypoxia or hypoxic mimetics induce expression of WNT11 in a wide variety of cell types

**Figure 1:**
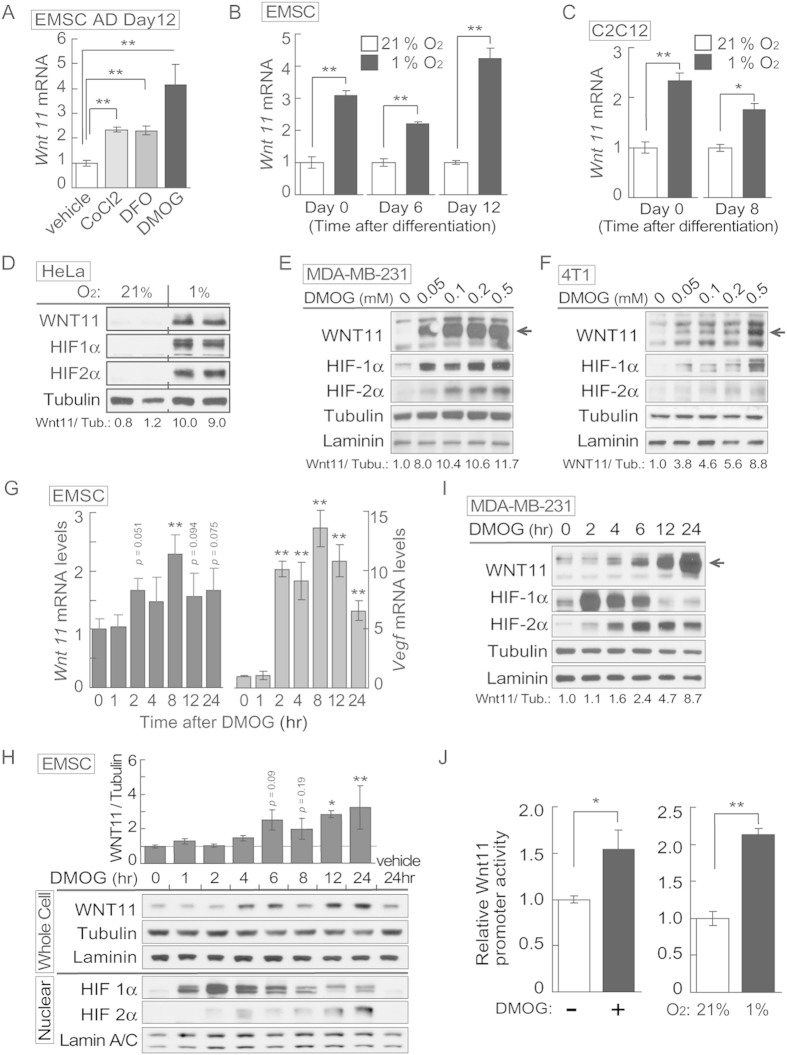
WNT11 is induced by hypoxia or *hypoxic mimetics* in different cell types. (**A**) Increased *Wnt11* mRNA in EMSC adipocytes (Day 12) after hypoxia-mimetic treatments. EMSC adipocytes were treated with CoCl_2_ (0.1 mM), DFO (0.1 mM) or DMOG (0.1 mM) for 24 hrs. Values were normalized to *Tbp* mRNA and are expressed relative to control (*n* = 3). (**B**,**C**) Increased *Wnt11* mRNA by hypoxia in EMSC preadipocytes and adipocytes (Day 0–12 after differentiation) (**B**), and C2C12 myoblast and myocyte (Day 0 and 8 after differentiation) (**C**). *Wnt11* mRNA was assessed by quantitative PCR in cells exposed to air (21% O_2_) or hypoxia (1% O_2_) for 24 hrs. (*n* = 4). Values were normalized to *Tbp* mRNA and are expressed relative to 21% O_2_ samples (left panel). (**D**) Immunoblot analyses of HeLa cells under normal air or hypoxia for 24 hrs. (**E**,**F**) Induction of Wnt11 by increasing concentrations of DMOG in MDA-MB-231 cells (**E**) and 4T1 cells (**F**). (**G**) EMSCs treated with 0.1 mM DMOG for the indicated times. *Wnt11* and *Vegf* mRNA expression was measured by qPCR and normalized to *Tbp* mRNA (*n* = 4). (**H**) WNT11 protein levels after DMOG treatment normalized to α-Tubulin (upper panel; *n* = 4). Representative immunoblots of EMSCs treated with 0.1 mM DMOG for the indicated times (Lower panel). (**I**) Protein expression in MDA-MB-231 cells treated with 0.1 mM DMOG. (**J**) Induction of Wnt11 promoter activity by hypoxia or hypoxia mimetics. pGL3-Wnt11 promoter plasmid was transfected into C2C12 cells. Cells were incubated with DMOG (left panel, *n* = 4) or under 21% O_2_ or 1% O_2_ (right panel, *n* = 8) for 24 hrs. For panels (**A**–**C**,**G**,**H**,**J**), values are mean ± s.e.m. *p < 0.05, **p < 0.01. For panels of immunoblotting, laminin, α-tubulin, and ERK were used as loading controls, WNT11 normalized to α-Tubulin was shown.

During a screen of Wnt genes regulated by hypoxia, we observed that mRNA of *Wnt11* was robustly and specifically induced by hypoxia mimetics, such as cobalt chloride CoCl_2,_ deferoxamine (DFO), and dimethyloxalylglycine (DMOG) in fully differentiated adipocytes (Fig. 1A). In contrast, expression of Wnt5a and secreted frizzled related protein-5 (Supplementary Figure 1A) were both decreased under these conditions. Moreover, culture of ear mesenchymal stem cells (EMSCs) or C2C12 cells at various stages of differentiation in 1% O_2_ increased Wnt11 (Fig. 1B,C and Supplementary Figure 1B,C). Interestingly, we also observed increased WNT11 protein in response to hypoxia or hypoxia-mimetic reagents in a macrophage cell line (Supplementary Figure 1D), and human cancer-derived cell lines (HeLa; Fig. 1D, MDA-MB-231; Fig. 1E). Expression of WNT11 protein was increased by DMOG in a concentration-dependent manner (Fig. 1E,F and Supplementary Figure 1E). Thus, induction of WNT11 appears to be a general response to hypoxia found in a wide variety of cell types.

To determine how rapidly hypoxia influences WNT11 expression, a time course after exposure of EMSCs to DMOG was assessed. Hypoxia increased *Wnt11* mRNA as early as 2 hr, and WNT11 protein at 4 hr (Fig. 1G–I; Supplementary Figure 1F). Two hours was also the first time point where increased *Vegf* mRNA was also detected, although the magnitude of induction was much larger in this case (Fig. 1G). VEGF is a well-characterized HIF target gene^7^, suggesting that elevated WNT11 may also be a direct target of HIF-1α and/or −2α. Following DMOG treatment of MDA-MB-231 cells, HIF-1α is rapidly and transiently induced whereas the elevation of HIF-2α is slightly delayed but more sustained (Fig. 1H,I)

To test whether effects of hypoxia to increase *Wnt11* mRNA are transcriptional, we next explored the possibility that hypoxia affects Wnt11 promoter activity. Bioinformatic analysis suggested the presence of two potential HIF-binding DNA elements in the Wnt11 promoter (Supplementary Figure 1G). To this end, 1064 bp upstream of the mouse Wnt11 transcription start site was cloned into a luciferase reporter vector. After transient transfection into C2C12 cells, we observed that Wnt11 promoter activity was induced by both DMOG and hypoxia (Fig. 1J), suggesting that regulation of *Wnt11* by hypoxia is mediated at the transcriptional level.

### Von Hippel-Lindau (VHL) represses expression of WNT11

**Figure 2:**
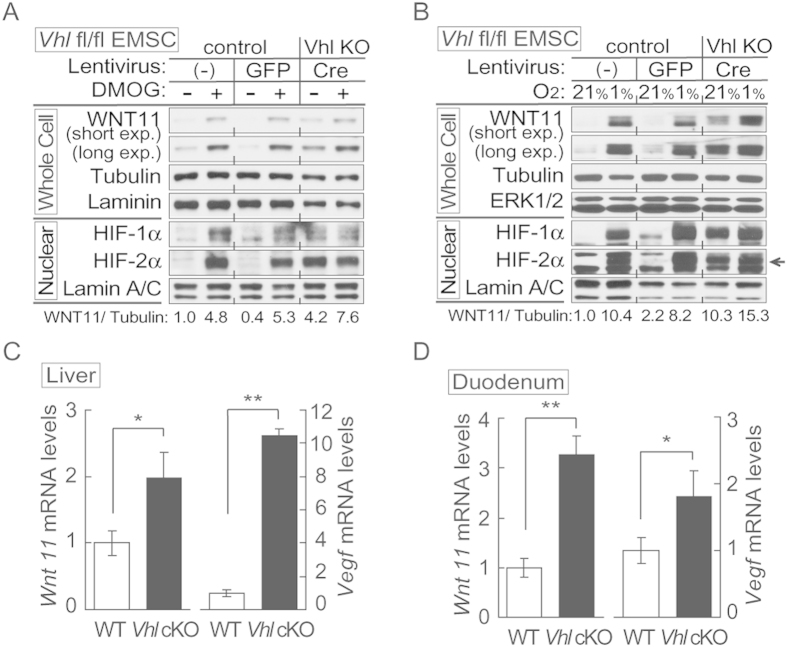
Hypoxia induces expression of WNT11 through VHL. (**A**,**B**) Higher basal levels of WNT11 protein in *Vhl*-deleted cells (lenti-Cre infected *Vhl*^f/f^). EMSCs isolated from *Vhl*^f/f^ mouse were infected with lentivirus carrying either GFP gene (for control) or Cre recombinase (for knockout). Non-infected cells were also used as a control. Immunoblot analysis of control or *Vhl* KO EMSCs treated with 0.1 mM DMOG (**A**), and EMSCs exposed to air (21% O_2_) or hypoxia (1% O_2_) for 24 hrs (**B**). Laminin, α-tubulin, and lamin A/C were used as loading controls, WNT11 normalized to α-Tubulin was shown. (**C**,**D**) Inactivation of the *Vhl* gene results in increased *Wnt11* mRNA. *Wnt11* and *Vegf* mRNA levels in liver (**C**) or duodenum (**D**) were measured by qPCR in *Liver-Vhl*cKO or *duodenum-VhlcKO* and control mice (*n* = 5 per group). Values normalized to *Tbp* mRNA are expressed relative to tissues from control mice. For panels (**C**,**D**), values are mean ± s.e.m. *p < 0.05, **p < 0.01.

Hypoxia is well known to activate gene transcription through the HIF pathways, but signaling through mechanisms independent of HIFs have also been described^26^. To determine if WNT11 induction by hypoxia is HIF-dependent, we first studied cells and tissues lacking the VHL protein, which forms part of an E3 ubiquitin ligase that targets HIF-1α/2α for degradation. To generate *Vhl*^−/−^ cells, EMSCs isolated from the *Vhl*^fl/fl^ mouse were infected with lentivirus carrying Cre recombinase (for *Vhl*^−/−^) or GFP (for control). *Vhl*^−/−^ EMSCs had increased amounts of HIF-1α, HIF-2α and WNT11 proteins compared to non-infected or GFP-infected control EMSCs under standard culture conditions (21% O_2_). The induction was similar to that observed with DMOG or 1% O_2_ (Fig. 2A,B). To study regulation of WNT11 by VHL, we analyzed mice lacking *Vhl* in specific tissues. Consistent with our observations on induction of WNT11 by hypoxia (Fig. 1), knockout of the tumor suppressor *Vhl* in liver or duodenum resulted in increased *Wnt11* mRNA (Fig. 2C,D), suggesting that the classical hypoxia signaling pathway regulates *Wnt11*.

### HIF-1α is an important transcriptional regulator of WNT11 expression

**Figure 3:**
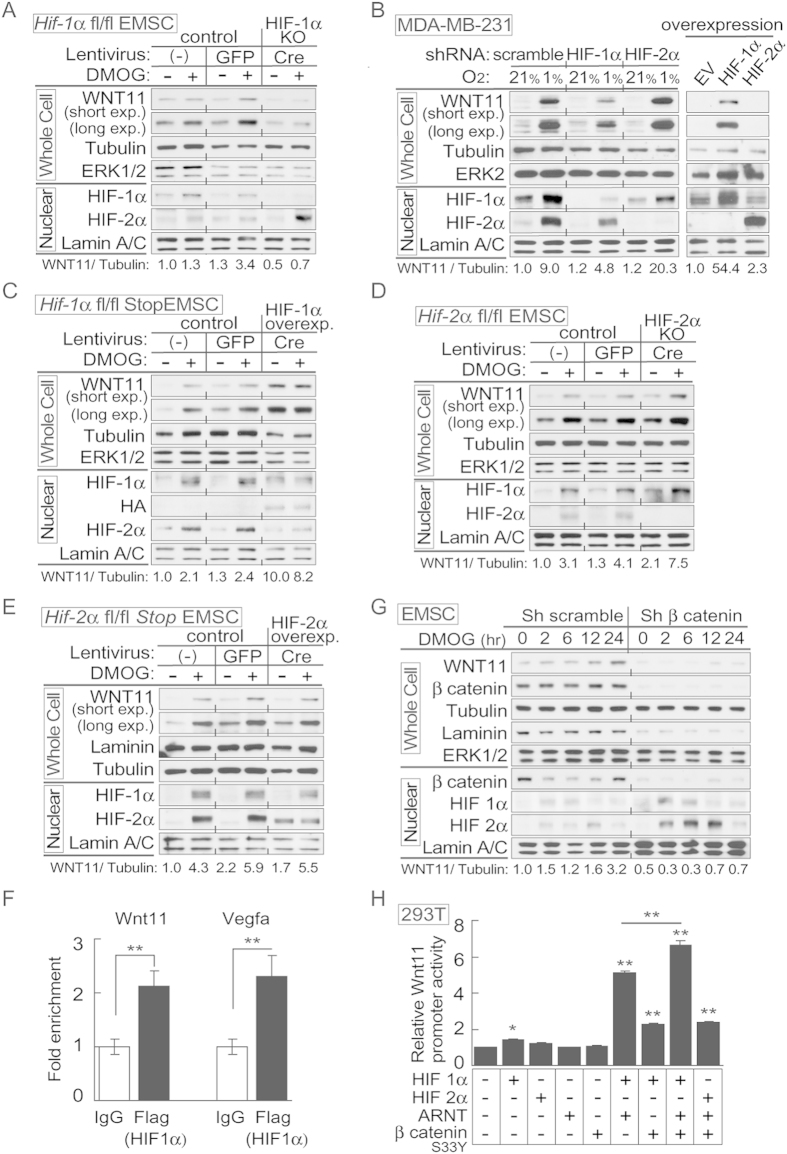
HIF-1α is the predominant transcriptional regulator of WNT11 expression during hypoxia. (**A**,**C**–**E**) EMSCs isolated from the indicated mouse genotypes were infected with lentivirus expressing GFP or Cre recombinase. Non-infected cells and GFP infected cells served as controls. Immunoblot analyses of EMSCs derived from the indicated genotypes treated with 0.1 mM DMOG for 24 hrs. (**A**) Attenuated WNT11 expression in *Hif-1α* KO EMSCs (lenti-Cre infected *Hif-1a*^f/f^). (**B**) HIF-1α regulates WNT11 expression during hypoxia. Impaired WNT11 expression in MDA-MB-231 cells stably expressing *HIF-1α* shRNAs with hypoxia. Cells were exposed to air (21% O_2_) or hypoxia (1% O_2_) for 24 hrs (left panel). Overexpression of *HIF-1α* in MDA-MB-231 cells enhances WNT11 expression (right panel). (**C**) Markedly increased WNT11 levels in *Hif-1α* overexpressing EMSCs (lenti-Cre infected *Hif-1α-Tg*^fl-Stop^). (**D**) Elevated HIF-1α and WNT11 protein after DMOG treatment in HIF-2α KO cells (lenti-Cre infectied *Hif-2α*^fl/fl^). (**E**) Little effect of HIF-2α overexpression on WNT11 protein expression in EMSC (lenti-Cre infectied *Hif-2α-Tg*^fl-Stop^). (**F**) HIF-1α binds to WNT11 and VEGF promoters as assessed by ChIP analyses (*n* = 3 each condition). (**G**) WNT11 expression was suppressed in cells with a β-catenin deficiency. EMSCs stably expressing shRNAs against β-catenin or scrambled control were treated with 0.1 mM DMOG for 24 hrs and analyzed by immunoblotting. (**H**) Co-transfection with expression vectors for β-catenin, Hif-1α and ARNT stimulates further induction of Wnt11 promoter activity. Constructs encoding Hif-1α, Hif-2α, ARNT, β-catenin and Wnt11 promoter-luciferase reporter, were transiently transfected into HEK293T cells. Cells were harvested and luciferase activities were measured 48 hrs after transfection (*n* = 3). For panels (**A**–**E**,**G**), laminin, α-tubulin, ERK1/2 and lamin A/C were used as loading controls, WNT11 normalized to α-Tubulin was shown under the bots. For panels (**F,H**), values are mean ± s.e.m. *p < 0.05, **p < 0.01

Activation of WNT11 by VHL disruption raised the possibility that HIF-1α and/or HIF-2α may regulate *Wnt11* expression. Through gain- and loss-of-function experiments the specific effects of HIF-1α and/or HIF-2α on Wnt11 expression under hypoxic conditions were assessed. EMSCs from *Hif-1α*^fl/fl^ or *Hif-2α*^fl/fl^ mice were infected with lentivirus carrying either the GFP gene (for control) or Cre recombinase (for knockout), and EMSCs from *Hif-1α-Tg*^fl-Stop^ or *Hif-1α-Tg*^fl-Stop^ mice were infected with lentivirus-GFP (for control) or Cre recombinase (for overexpression). As shown in Fig. 3A, WNT11 was induced by DMOG treatment in control cells (non-infected cells or cells infected with lenti-GFP), but both basal and DMOG-induced WNT11 expression were markedly attenuated in *Hif-1α* KO EMSCs (Fig. 3A). Similarly, induction of WNT11 expression in MDA-MB-231 cells by hypoxia was reduced by stable expression of *HIF-1α* shRNAs (Fig. 3B; left panel). Conversely, a marked increase in WNT11 levels were observed in *Hif-1α* overexpressing EMSCs (*Hif-1α-Tg*^fl-Stop^ EMSC infected with lenti-Cre) under normoxic conditions and with DMOG treatment (Fig. 3C), and overexpression of *HIF-1α* in MDA-MB-231 cells (Fig. 3B; right panel). Consistent with previous reports^27^, we observed a reciprocal relationship between HIF-1α and HIF-2α expression: HIF-2α expression was elevated in HIF-1α knockout cells in response to hypoxia (Fig. 3A) and HIF-2α levels were decreased with HIF-1α overexpression (Fig. 3C). These data suggest that HIF-1α is both necessary and sufficient to regulate WNT11 expression.

We next evaluated the potential regulation of WNT11 by HIF-2α, and found that knockout of HIF-2α was associated with elevated WNT11 under basal and DMOG-induced conditions; however, both effects are likely secondary to compensatory increases in HIF-1α protein levels (Fig. 3D). Consistent with regulation of WNT11 residing predominantly with HIF-1α, overexpression of HIF-2α had little effect on WNT11 protein level (Fig. 3B; right panel and 3E). HIF-1α binds to hypoxia response elements (HREs) of target genes as a heterodimer with ARNT^1^,^2^,^3^,^4^,^6^; thus, we next considered whether ARNT also regulates *Wnt11* transcription. Co-expression of HIF-1α and ARNT markedly induced *Wnt11* promoter reporter activity, whereas expression of either protein separately, or co-expression of HIF-2α and ARNT had only minor effects (Supplementary Figure 2A,B). To explore promoter occupancy of HIF-1α in the regulation of Wnt11 expression, HIF-1α chromatin immunoprecipitation analysis was performed and we found that Flag-HIF-1α binds to the Wnt11 promoter in MDA-MB-231 cells (Fig. 3F). As a positive control, the VEGFα promoter was also specifically enriched by Flag-HIF-1α ChIP (Fig. 3F). These physical interactions provide a mechanistic basis for transactivation of Wnt11 promoter by HIF-1α. Taken together, these data provide evidence that Wnt11 expression is primarily regulated by VHL, HIF-1α and ARNT.

WNT/β-catenin signals regulate WNT11 expression in a variety of cell types and species^12^. To evaluate whether β-catenin is required for hypoxia-induced WNT11 expression, we generated EMSCs with stable expression of shRNAs against β-catenin. Whereas DMOG treatment caused a transient decline in β-catenin, basal WNT11 and expression of WNT11 after DMOG treatment was strongly suppressed in cells with a β-catenin deficiency (Fig. 3G). Consistent with data presented above, increased HIF-2α with β-catenin knockdown is not sufficient to elevate WNT11 (Fig. 3G). Although hypoxia did not induce accumulation of nuclear β-catenin (Supplementary Figure 2C), β-catenin is essential for WNT11 expression, in accordance with the previous reports that β-catenin regulates WNT11 expression in other contexts^12^. Furthermore, we observed additive induction effects on *Wnt11* promoter activity when β-catenin was co-transfected with HIF-1α and ARNT expression vectors (Fig. 3H). Taken together, these data indicate that HIF-1α and β-catenin are required for WNT11 expression.

### WNT11 is important for cell migration and invasion

**Figure 4:**
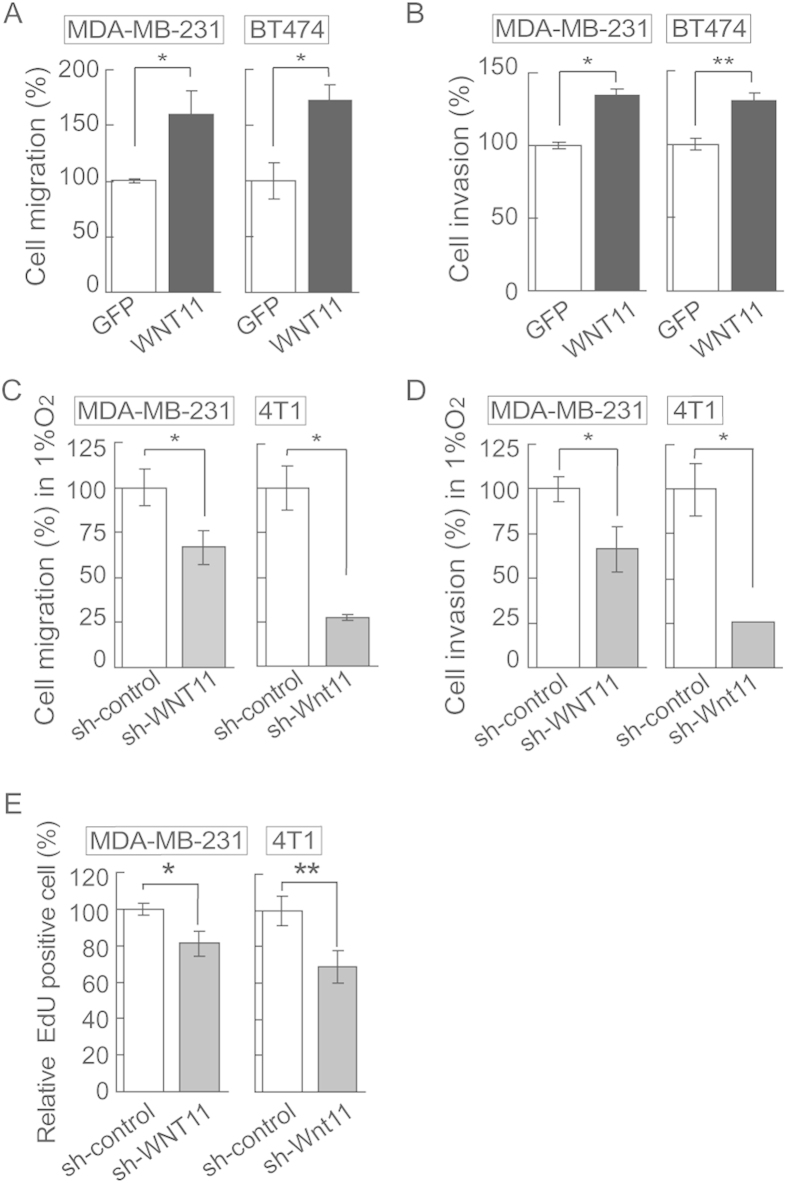
WNT11 increases cancer cell migration and invasion. (**A**,**B**) Stable overexpression of WNT11 enhances migration (**A**) and invasion (**B**). Boyden chamber assays were used to analyze migration of MDA-MB-231 and BT-474 cells infected with lentiviruses for stable expression of Wnt11 or GFP (*n* = 3). (**C**,**D**) MDA-MB-231 cells and 4T1 cells stably expressing shRNAs against WNT11 showed impaired migration (**C**) and invasion (**D**) under hypoxia. Cells were incubated with 0.2 μM DMOG for 8 hrs before seeding on the chamber to induce WNT11 expression. Cells in Boyden chamber were then incubated under hypoxic conditions (1% O_2_) (*n* = 3). (**E**) Loss of Wnt11 inhibits 4T1 (*n* = 6) and MDA-MB-231 (*n* = 5) cell proliferation. EdU-positive cells among DAPI-positive cells were quantified. Data are expressed as the percentage of their respective scrambled control cells. Values are mean ± s.e.m. *p < 0.05, **p < 0.01.

HIF-1α has extensive direct and indirect effects on gene expression^2^,^3^,^5^, and a subset of HIF-1α targets play important roles in diverse aspects of cancer biology, including migration and invasion^28^,^29^,^30^, and metastasis^31^,^32^,^33^. The induction of WNT11 by hypoxia, and the reported ability of WNT11 to influence cell mobility^15^,^20^,^21^,^22^,^34^,^35^,^36^ led us to hypothesize that WNT11 plays an intermediary role in the effects of HIF-1α on cell migration and invasion. To understand effects of WNT11 on cancer cells, we generated stable overexpression of WNT11 in BT-474 cells (Supplementary Figure 3A), which have amongst the lowest expression of WNT11 in cell lines tested (Supplementary Figure 3B). We observed that ectopic expression of WNT11 facilitates both migration and invasion of these cells *in vitro* (Fig. 4A,B). We then examined the functional consequences of endogenous WNT11 induced by hypoxia. WNT11 was knocked down with lentiviral shRNAs in 4T1 or MDA-MB-231 cells, the latter of which have the highest endogenous expression of WNT11 in the cancer cell lines tested (Supplementary Figure 3B–D). Whereas reduced expression of WNT11 did not influence cell mobility under normoxic conditions (Supplementary Figure 4A,B), impaired migration (Fig. 4C) and invasion (Fig. 4D) were observed in WNT11 knockdown cells when cells were incubated under hypoxic conditions. Furthermore, we confirmed impaired migration and invasion in cells with CRISPR-Cas9-based knockout of *WNT11* (Supplementary Figures 3E,F and 4C,D). Consistent with previous reports^12^,^15^,^22^,^37^, effects of WNT11 on cell growth are through cell proliferation (Fig. 4E), although differences in cell number of endogenous Wnt11 depletion are not easily detected until 48 hr after initial plating of MDA-MB-231 or 4T1 cells under normoxia (Supplementary Figure 4E,F). Although DMOG had no effect on cell growth during this time frame, incubation in 1% O_2_ decreased growth rate of both cell types (Supplementary Figure 4E,F). To minimize potential effects of differential growth rates on migration and invasion, we optimized plating numbers and used short incubation times during which effects of WNT11 on number of viable cells by MTT assay were not observed (Supplementary Figure 4G,H). These results confirm previous reports that WNT11 functions to increase mobility of cancer cells^15^,^22^, and demonstrate the intermediary role of WNT11 in regulation of migration and invasion by hypoxia.

### WNT11 regulates MMP activity

**Figure 5:**
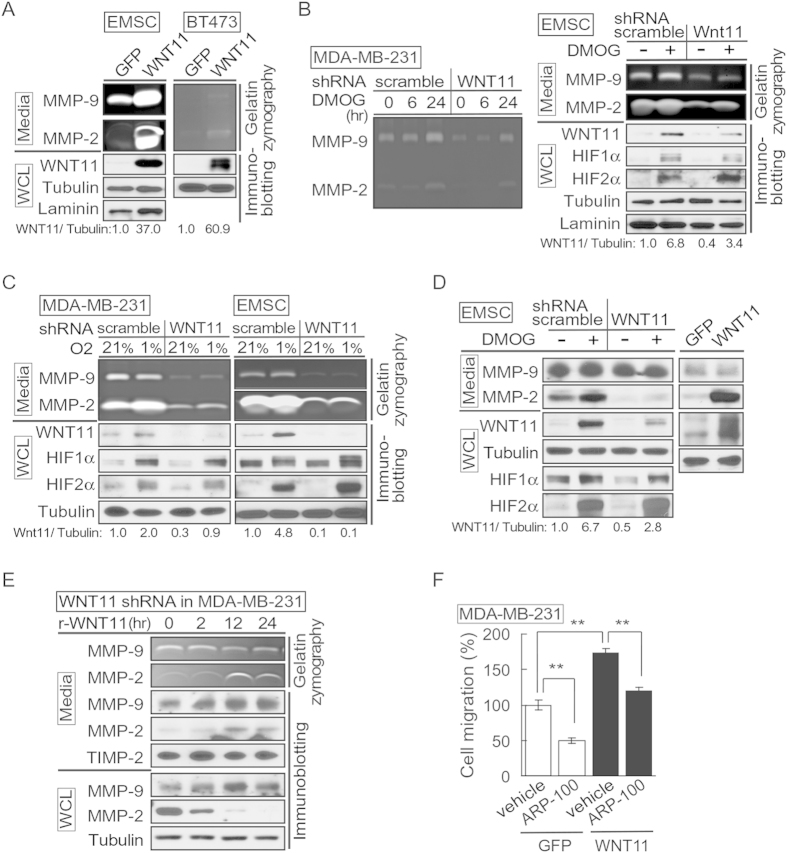
WNT11 regulates MMPs activities. (**A**–**C**) (Top panels): Serum-free medium was conditioned for 24 hrs by the indicated cells, concentrated 20-fold and assayed by gelatin zymography. Gelatinolytic activity is indicated by clear zones against a dark background of stained substrate. (Bottom): Whole cell extracts were immunoblotted with indicated antibodies. (**A**) Overexpression of Wnt11 in EMSC or BT473 cells enhances activity of MMP-9 and MMP-2. (**B**) Impaired activity of MMP-9 and MMP-2 in MDA-MB-231 cells (left) or EMSCs (right) stably expressing *Wnt11* shRNAs and treated with DMOG. (**C**) WNT11 is required for MMP-9 and MMP-2 activity in MDA-MB-231 cells (left) or EMSCs (right) under normoxic and hypoxic culture conditions. (**D**) WNT11 regulates MMP2 protein in media. (Top): conditioned media from indicated cells and treatments. (Bottom): whole cell lysates were immunoblotted with indicated antibodies. (**E**) Recombinant WNT11 induces both MMP-2 protein and MMP-2 activity in media. (Top panels): Gelatin zymography and immunoblot of serum-free medium conditioned for the indicated times after recombinant WNT11 (r-WNT11) treatment. (Bottom): Whole cell lysates were immunoblotted with indicated antibodies. (**F**) MMP-2 inhibitor attenuated induced migration by WNT11. MDA-MB-231 cells infected with lentiviruses for stable expression of Wnt11 or GFP (*n* = 4) were incubated with either vehicle or 1 μM of ARP100. Media in the lower compartment had same concentration of DMSO or inhibitor. Values are mean ± s.e.m. *p < 0.05, **p < 0.01. For panels (**A**–**D**), HIF-1α and HIF-2α were shown as a marker of hypoxia, WNT11 normalized to α-Tubulin was shown.

To determine the mechanism by which WNT11 regulates migration and invasion, we evaluated MMP activities using zymography. HIF-1α and β-catenin, two factors we describe as upstream of WNT11, both regulate mRNA and/or enzyme activity of MMP-2 and MMP-9^28^,^38^,^39^,^40^. Overexpression of WNT11 in EMSCs and BT-474 breast cancer cell lines resulted in higher MMP-2 and/or MMP-9 zymographic activities (Fig. 5A). To explore potential roles of endogenous WNT11 in modulation of MMP activities, we used shRNA to block induction of WNT11 by hypoxia. In MDA-MB-231 cells, DMOG increased MMP-9 and MMP-2 activity between 6 and 24 hrs after treatment, and knockdown of *WNT11* decreased basal and DMOG-induced MMP activities (Fig. 5B; left panel). Knockdown of *Wnt11* also decreased baseline and DMOG-induced WNT11 in EMSCs without influencing induction of HIF-2α (Fig. 5B; right panel). Whereas hypoxia did not increase MMP-9 activity in MDA-MB-231 (Fig. 5C; left panel) or EMSCs (Fig. 5C; right panel), increases in MMP-2 activity were observed in both cell models. Knockdown of *WNT11* decreased WNT11 and both MMP activities under standard and hypoxic cell culture conditions (Fig. 5C). We further determined whether altered MMP activity was explained by amount of MMP protein. Interestingly, we found decreased MMP-2 protein level in media corresponding to decreased WNT11 expression, and a reciprocal change arising from WNT11 overexpression (Fig. 5D). No change in MMP-9 protein levels was observed even when MMP-9 activity was regulated by WNT11 expression (Fig. 5A–C), suggesting regulation through other factors such as tissue inhibitor metalloproteinases (TIMP) and/or cell surface activator of proMMPs (MT-MMP). Furthermore, recombinant human WNT11 induced both MMP-2 protein and activity in the media of *WNT11* knockdown cells (Fig. 5E) with decreased MMP-2 in cell lysates (Fig. 5E), suggesting that hypoxia-WNT11 pathway regulates MMP2 expression and secretion. In addition, MMP-2 inhibitor (ARP-100) suppressed migration of MDA-MB-231 cells expressing both GFP and exogenous WNT11 (Fig. 5F). Taken together, our results suggest that in response to hypoxia, elevated WNT11 promotes migration and invasion by increasing the activity of MMP-2 and MMP-9.

### WNT11 is induced by hypoxia and regulates tumor cell growth *in vivo*

**Figure 6:**
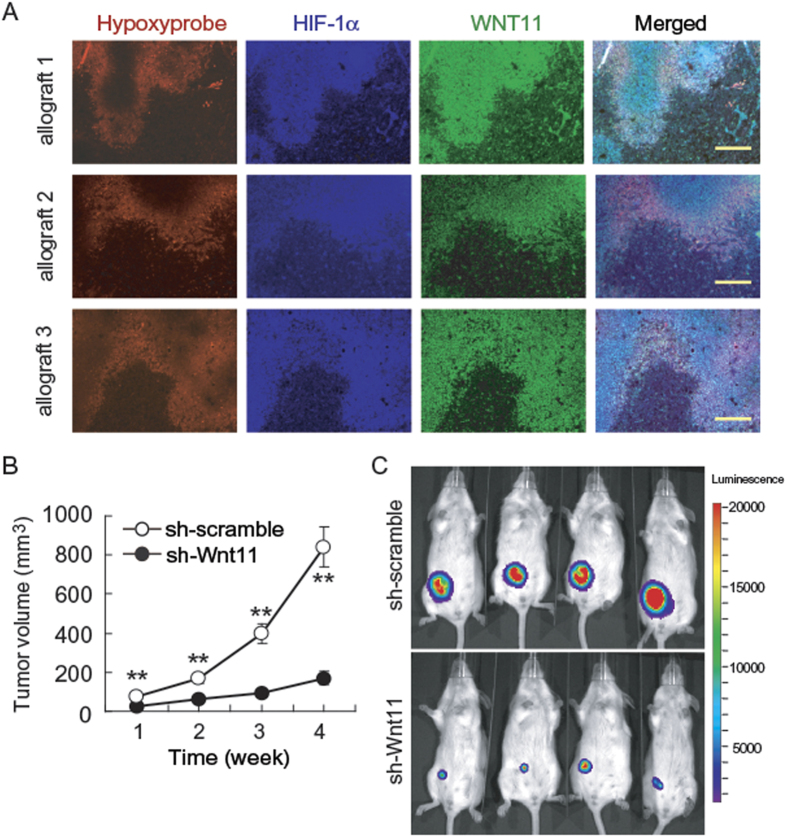
Induced WNT11 expression with tumor hypoxia and WNT11 regulates tumor growth. (**A**) WNT11 is elevated in hypoxic regions of tumors. Mammary pads of female BALB/c mice were injected with 4T1 cells. Five weeks after transplantation, tumor tissue was harvested and pimonidazole (Hypoxyprobe; red), HIF-1α (blue), Wnt11 (green) and their colocalization (right) in tumor tissue are shown. The scale bar represents 200 μm. (**B**) Wnt11 deficiency suppresses the progression of 4T1 breast cancer in mice. 4T1 cells stably expressing shRNAs against Wnt11 or scrambled control were injected to BALB/c mice, and then tumor size was measured (*n* = 8). Values are mean ± s.e.m. **p < 0.01. (**C**) Bioluminescence imaging of tumor bearing mice. Representative images taken two weeks after injection of firefly luciferase-tagged 4T1 cells stably expressing shRNAs against Wnt11 or scrambled control into BALB/c mice.

To extend our results with cultured cells, we investigated whether WNT11 is expressed in hypoxic regions of allografts. After validation of the WNT11 antibody (Supplementary Figure 5), we observed that WNT11 is elevated in the area of tumor hypoxia where HIF-1α and pimonidazole staining are colocalized (Fig. 6A). In addition, we observed decreased tumor growth in mice injected with 4T1 cells expressing shRNAs against *Wnt11* (Fig. 6B,C), and reduced proliferation in WNT11 knockout cells *in vitro* (Fig. 4E), suggesting that impaired tumor progression in Wnt11 shRNA allograft is secondary to reduced proliferation. These observations are consistent with previous reports that WNT11 plays a key role in tumor progression^12^,^15^. Although these data are interesting, the impaired growth of *Wnt11* knockdown cells precluded evaluation of metastasis or effects of antiangiogenic treatments.

**Figure 7:**
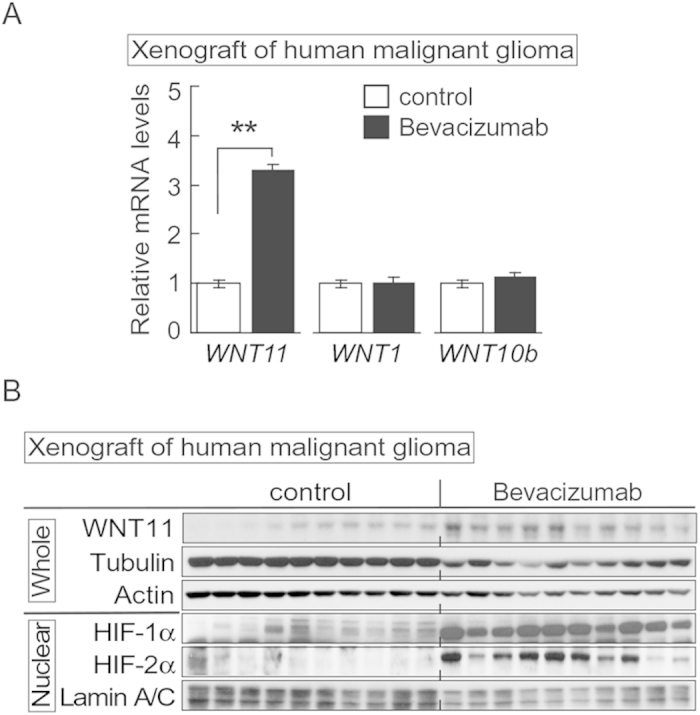
Induced WNT11 expression with tumor hypoxia and WNT11 regulates tumor growth. Antiangiogenic therapy induces WNT11 expression in the orthotopic malignant glioma model. Athymic mice implanted with U87ΔEGFR cells were administered either bevacizumab (6 mg/kg) or vehicle three times per week for 4 weeks. (**A**) Increased Wnt11 mRNA in xenografts from mice treated with bevacizumab. Values were normalized to HPRT mRNA and are expressed relative to control (*n* = 10 per group). Values are mean ± s.e.m. *p < 0.05, **p < 0.01. (**B**) Bevacizumab increased expression of HIF-1α and HIF-2α and WNT11. First 10 lanes are control tumors, and the last 10 lanes are tumors from bevacizumab-treated animals. Lysates from whole tissue and nuclei are indicated. α-Tubulin, actin and lamin A/C are loading controls.

As another *in vivo* model, we chose to evaluate malignant gliomas because these tumors secrete high levels of VEGF and are refractory to bevacizumab, a monoclonal antibody to inhibit VEGF^23^,^25^. Using an orthotopic malignant glioma model in which U87ΔEGFR cells are injected into the frontal lobe of athymic mice, we observed increased expression of WNT11 mRNA in animals treated with bevacizumab (Fig. 7A). However, we saw no effects on WNT1 and WNT10b (Fig. 7A), which were recently reported to be induced by hypoxia-HIF-2α in adipogenic cells^41^. Furthermore, bevacizumab treatment stimulated expression of WNT11 protein, as well as nuclear HIF-1α and HIF-2α (Fig. 7B). These data provide further evidence that WNT11 is induced by hypoxia *in vivo*, and raise the possibility that induction of WNT11 is involved in hypoxia-induced metastatic and invasive potential of tumor cells.

## Discussion

This study provides the first demonstration that WNT11 expression is regulated by hypoxia and the HIF-1α pathway in normal and cancer-derived cells (Supplementary Figure 6). Previous studies have identified factors that directly or indirectly regulate WNT11 expression, including glial cell-line derived neurotrophic factor signaling and estrogen/ERR-α^12^ (Supplementary Figure 6). Signaling by the Wnt/β-catenin pathway also enhances expression of WNT11, consistent with two conserved Tcf/LEF binding sites in the Wnt11 promoter^12^, and our results support a β-catenin requirement for the induction of WNT11 (Fig. 3G,H). Relationships between hypoxia and Wnt signaling have been explored in neural stem cells where HIF-1α enhances β-catenin activation and expression of downstream effectors LEF-1 and TCF-1^9^. Hypoxia causes nuclear accumulation of both HIF-1α and β-catenin in induced pluripotent stem cells^42^. We did not observe accumulation of nuclear β-catenin in cells under hypoxia or DMOG treatment (Fig. 3G, Supplementary Figure 2C), and other investigators have observed that hypoxia suppresses β-catenin nuclear localization and/or TOP-Flash activity^8^,^43^. Thus, effects of hypoxia on β-catenin appear to be cell type dependent.

Hypoxia is an important regulator of cell migration and invasion under physiological and pathological conditions^28^,^29^,^30^,^31^,^32^,^33^. In the hypoxia associated with cancer, HIF-1α and a subset of its targets play important roles in migration, invasion^28^,^29^,^30^, and metastasis^31^,^32^,^33^. Our finding that expression of WNT11 is induced by hypoxia/HIF-1α is of particular interest because WNT11 increases cell migration/invasion during both development^15^,^20^,^21^,^34^,^35^,^36^ and carcinogenesis^22^. In line with these findings, loss- and gain-of-function experiments reveal that WNT11 plays an essential role in triggering cell migration and invasion (Fig. 4, Supplementary Figures 4 and 6). Recent studies using time-lapse microscopy revealed that at least 14 hours were required for breast cancer cells to acquire enhanced motility under hypoxic conditions^44^. Our experiments are consistent with this timeframe, and cells required pretreatment with DMOG before the migration and invasion assay in order to induce WNT11 expression (Figs 1G–I and 4C,D). Interestingly, stimulation of RhoA and Rock by HIFs is important for regulation of cell motility under hypoxia^44^; thus, the observation that WNT11 also induces activation of RhoA is consistent with an intermediary role^22^. It should be noted however that another report suggests a tumor suppressor role for WNT11 in hepatocellular carcinoma cells^37^.

Inhibiting key players in tumor angiogenesis such as VEGF and VEGF receptor^7^ holds significant potential for treatment of cancers; however, a limited number of patients respond to antiangiogenic agents. Furthermore, this therapy may in some cases increase tumor invasiveness and metastasis^23^,^24^,^25^. For example, anti-VEGF treatment of malignant glioma appears to have therapeutic benefit in patients, but long-term outcomes have not been improved^45^, perhaps because treatment induces a phenotypic shift towards more aggressive forms^46^. Whereas tumor hypoxia following antiangiogenic treatment is proposed as the cause of poor outcome, little is known about the underlying mechanisms. Although elevated WNT11 was not observed in tumors derived from glioblastoma spheroids from two patients implanted in rat^23^ (data not shown), we tested effects of bevacizumab in an orthotopic malignant glioma mouse model, and found that anti-VEGF treatment increased HIF-1α and HIF-2α, and also induced expression of *WNT11* mRNA and protein (Fig. 7). The effects of antiangiogenic therapy in our study appear to be specific to WNT11 in that we did not see effects on WNT1 and WNT10b, which are induced by hypoxia and HIF-2α in adipogenic cells^41^. Further work will be required to evaluate whether elevated WNT11 is necessary or sufficient to cause the mesenchymal transition associated with long-term antiangiogenic therapy of glioma tumor tissue *in vivo*^46^. We also observed that growth of Wnt11 deficient allografts was significantly slower (Fig. 6B,C). In addition, we performed metabolomic experiments using conditioned media from Wnt11 knockdown and control 4T1 cells. As often occurs in proliferating cancer cells in the presence of oxygen^47^, we observed that control 4T1 cells consumed more fuel and amino acids and produced more lactic acid than Wnt11 knockdown cells (data not shown). It may be interesting if induction of WNT11 by tumor hypoxia protects against cell death, as was observed in cardiomyocytes^48^,^49^. Taken together, these data suggest WNT11 as a possible target for cancer therapies, especially with tumor hypoxia and/or tumors treated with antiangiogenic therapy. Blocking WNT11, either by inhibiting upstream regulators (HIFs and/or β-catenin) or with WNT11 neutralizing antibody^48^,^49^ can be a novel therapeutic strategy. Further studies will be needed to examine effectiveness of this treatment.

## Materials and Methods

### Cell Culture

Ear mesenchymal stem cells (EMSC) were isolated from the outer ears of C57BL/6J mice as well as the *Vhl*^f/f^, *Hif-1*α^f/f^, *Hif-2*α^f/f^, *Hif-1*α*-Tg*^fl-Stop^ lines^50^ and maintained as previously described^51^. Isolated EMSC from *Vhl*^f/f^, *Hif-1*α^f/f^, *Hif-2*α^f/f^, *Hif-1*α*-Tg*^fl-Stop^ and *Hif-2*α*-Tg*^fl-Stop^ mice were infected with lentivirus carrying either the GFP gene or the Cre recombinase. EMSCs were used before differentiation unless otherwise indicated. C2C12 (ATCC) myogenesis was as described previously^50^. Culture medium was high-glucose (4.5 mg/mL) DMEM (GIBCO) for MDA-MB-231 (ATCC), BT-474 cells (ATCC), and human glioma cell line U87ΔEGFR^47^, and RPMI1640 medium for 4T1-luc cells (ATCC), supplemented with 10% FBS and penicillin–streptomycin. For hypoxia experiments, cells were incubated in 1% O_2_ and 5% CO_2_ and 94% N_2_ at 37 °C using hypoxia incubator chamber (STEMCELL Technologies).

### Migration, invasion and zymography assay

One day prior to seeding for both assays, cells below 80% confluence were incubated in reduced serum media (1% FBS) for 16 hrs, and then in 0.1% FBS media for 8 hrs. For migration assays, cells were seeded onto Transwell with 8.0 μm Pore Polycarbonate Membrane Inserts (Corning). The optimal seeding density and incubation time at 37 °C (5% CO_2_) was determined in preliminary experiments to be 3.3 × 10^4 ^cells per well for MDA-MB-231 and 4T1 cells (12 hr), and 1.2 × 10^5^ cells for BT-474 cells (16 hr). For invasion assays, Matrigel Invasion Chambers (BD Biosciences) were used according to the manufacturer’s instruction. Similarly, the optimal seeding densities and incubation times were determined to be 5.0 × 10^4 ^cells per well for MDA-MB-231 and 4T1 cells (14 hr), and 1.4 × 10^5^ cells for BT-474 cells (18 hr). Transwells were subsequently fixed with 3.7% formaldehyde for 10 min and stained with 0.05% crystal violet for 30 min. Cells were manually counted by a blinded experimenter. Gelatin zymography was performed as previously described^52^,^53^,^54^.

### EdU cell proliferation assay

Twelve hours after plating on the chambered cover glass (Thermo Scientific Nunc), cells were labeled with EdU (10 μM, 30 min), then fixed, permeabilized, and click-labeled with an azide dye following manufacturer’s instructions (Click-iT EdU Alexa Fluor Cell Proliferation Assay kit; Life Technologies).

### Immunoblot analysis

Tissue or cell extracts were immunoblotted with antibodies specific for WNT11 (ab31962, Abcam), Wnt11 (#AF2647; R&D Systems), HIF-1α (NB100–105, NB100–134), HIF-2α (NB100–122), Laminin (Novus Biologicals), ERK, β-actin (Cell Signaling), β-catenin (BD BioScience), Lamin A/C (Santa Cruz Biotechnology), α-tubulin (Sigma-Aldrich), MMP-2 (IM33), MMP-9 (IM37) (EMD Millipore), and HA (Covance, Princeton, NJ). For detection of HIF-1α and HIF-2α, nuclear proteins were isolated using the NE-PER nuclear extraction kit according to manufacture’s protocol (Thermo Scientific). Quantification of WNT11, Tubulin or ERK protein expression were done using ImageJ software (NIH).

### Wnt11 luciferase reporter assay

A 1064bp DNA fragment in mouse Wnt11 promoter proximal region was cloned into XhoI/HindIII sites on pGL3-basic plasmid. HEK293T or C2C12 cells were transfected with Wnt11 luciferase plasmid and pRL Renilla Luciferase Vector, used as an internal control. Relative cellular luciferase activities were examined using the Dual-Luciferase Reporter Assay System (Promega, Madison, WI), and the luciferase signals were recorded with a Wallac Victor 1420 Plate Reader (Perkin-Elmer). The reporter activity was expressed as arbitrary luciferase units (firefly/renilla). Three independent experiments were carried out.

### ChIP assay

MDA-MB-231 human breast cancer cells were transfected with pcDNA or pcDNA-Flag-HIF-1α using Lipofectamine^®^ 3000 (Life Technologies). Forty-eight hours post transfection, cells were cross-linked using 1% formaldehyde for 15 minutes, and nuclei isolated from cells were sonicated for 20 minutes using Qsonica sonicator (Fisher Scientific). Chromatin was immunoprecipitated overnight using a monoclonal Flag-M2 antibody (Sigma). Decrosslinked DNA were digested with proteinase K and purified using PCR purification kit (Qiagen). Eluted DNA was amplified with qPCR using primers listed below. huWnt11 (−515 to −263 bp) sense 5′-GAATTGCCCCAGCTTACTGA-3′, antisense 5′-GACACAGCGAGAGGGAGAAG-3′. huVEGF sense 5′-GCCTCCCCCTTTGGGTTT-3′, antisense 5′-huVEGF 5′-GAGGGAAGAGGACCTGTTGGA-3′.

### Plasmids

Lenti-Cre (LV-Cre pLKO.1) and LV-GFP plasmids were purchased from Addgene (Cambridge, MA). pcDNA3-HIF1α, HIF2α, ARNT, pcDNA3-β-catenin S33Y have been described previously^55^,^56^. For stable expression of WNT11 in EMSC and BT-474 cells, mouse WNT11 was cloned into lentivirus expression vector pLKO.1. For this purpose, pcDNA3.1/Wnt11-mycHis plasmid kindly provided by Dr. Lynn Megeney (Ottawa Hospital Research Institute) was used as template to clone mouse Wnt11 coding sequence by PCR. To prepare the vector, Cre coding sequence was cut out from LV-Cre pLKO.1 plasmid and mouse Wnt11 coding sequence was ligated in by using XbaI/KpnI sites. The plasmid was sequenced to ensure the validity of the DNA sequence. Both human and mouse WNT11 were stably knocked down by expression of an shRNA from the pLKO.1 puro vector. The shRNA was designed according to the manufacturer’s instructions to target the following sequence of mouse WNT11: 5′-GGATGTGGGAGTTACAGAAAT-3′, human WNT11: 5′-TGTGGAAGCTACAGAAATA-3′, and β-catenin: GCACACGAATGGATCACAA.

### Reagents

Recombinant human WNT11 was purchased from R&D Systems (Minneapolis). DMOG (Cayman Chemical) was used at a concentration of 0.1 mM unless otherwise indicated. ARP-100 was purchased from Cayman Chemical, DFO and cobalt chloride were from Sigma-Aldrich. Bevacizumab was generously provided by Genentech (San Francisco, CA)/Roche (Basel, Switzerland)/Chugai Pharmaceutical Co (Tokyo, Japan).

### mRNA quantification by RT-PCR

RNA isolation, reverse transcription and Quantitative PCR were performed as previously described^51^. Primer sequences for real-time RT-PCR for mouse were: *Wnt11*-No1 sense 5′-CAAGTTTTCCGATGCTCCTATGAA-3′, antisense 5′-TTGTGTAGACGCATCAGTTTATTGG-3′3′, *Wnt11*-No2 sense 5′-CTCAAGACCCGCTACCTGTC-3′, antisense 5′-ACCACTCTGTCCGTGTAGGG-3′, *Vegfa* sense 5′-CCACGTCAGAGAGCAACATCA-3′, antisense 5′-TCATTCTCTCTATGTGCTGGCTTT-3′, Gene expression of each mRNA level was normalized TATA box-binding protein (*Tbp*), and hypoxanthine phosphoribosyltransferase 1 (*Hprt*), and the primers were described previously^51^. Primers for human were: *WNT11*-No1 sense 5′-TTCCGATGCTCCTATGAAGG-3′, antisense 5′-AGACACCCCATGGCACTTAC-3′, *WNT11*-No2 sense 5′-GCCAATAAACTGATGCGTCTACA-3′, antisense 5′-GTATCGGGTCTTGAGGTCAGC-3′, *WNT1* sense 5′-CGATGGTGGGGTATTGTGAAC-3′, antisense 5′-CCGGATTTTGGCGTATCAGAC-3′, *WNT10B* sense 5′-GTGAGCGAGACCCCACTATG-3′, antisense 5′-CACTCTGTAACCTTGCACTCATC-3′, *HPRT* sense 5′-CCTGGCGTCGTGATTAGTGAT-3′, antisense 5′-AGACGTTCAGTCCTGTCCATAA-3′.

### Animal study

All animal procedures were approved by the University of Michigan Committee on the Use and Care of Animals, or were in accordance with the approved guidelines of the Animal Research Committee of Okayama University. Brain xenograft was essentially as described previously^25^. Briefly, 2 μl of the U87ΔEGFR cell suspension (1.0 × 10^5^/μl) was injected into the right frontal lobe of athymic mice (BALB/c-nu/nu; CLEA Japan). Mice were randomly assigned to two groups, and PBS or bevacizumab (6 mg/kg) were administered three times per week, intraperitoneally, starting on day 5 after tumor cell implantation. Animals were sacrificed 18 days after implantation and tumors dissected for analyses.

For the syngeneic breast cancer model, 4T1 cells derived from BALB/c mammary tumors were grown to 80% confluence, were trypsinized, mixed with Matrigel 1:1 by volume (BD Bioscience), and injected into the fourth inguinal mammary pad of 10-week old female mice (5.0 × 10^5^ cells). Tumor size was measured by digital calipers, and tumor volume was calculated as (4/3) × π × (L/2) × (L/2) × (H/2). Five weeks after tumor cell injection, mice were euthanized, and tumor tissue harvested immediately and snap-frozen in liquid nitrogen. Hypoxia was detected using Hypoxyprobe following the manufacturer’s protocol (Natural Pharmacia International). We monitored invasion and metastasis by bioluminescent imaging using the IVIS Lumina Imaging System (Xenogen) after luciferin (Promega) injection into mice.

### Statistical analyses

All data are presented as mean ± s.e.m. and were analyzed by 2-tailed Student’s *t*-test or analyses of variance (ANOVA). The differences were considered to be significant if *p* < 0.05. Whereas a power equation was not used to predetermine sample sizes, our sample sizes were chosen to be similar to those reported in previous publications for *in vitro*^12^,^13^,^15^,^20^,^21^,^22^ and animal studies^23^,^24^,^25^ of this nature. No animals were removed from analyses.

## Additional Information

**How to cite this article**: Mori, H. *et al.* Induction of WNT11 by hypoxia and hypoxia-inducible factor-1a regulates cell proliferation, migration and invasion. *Sci. Rep.* **6**, 21520; doi: 10.1038/srep21520 (2016).

## Supplementary Material

Supplementary Information
