# Supplementary material for: Induction of WNT11 by hypoxia and hypoxia-inducible factor-1α regulates cell proliferation, migration and invasion
Source: Sci Rep. 2016 Feb 10;6:21520. doi: 10.1038/srep21520 (PMC4748282; doi:10.1038/srep21520)
Supplement: Supplementary Information [file srep21520-s1.pdf]

Supplementary Data

Induction of WNT11 by hypoxia and hypoxia-inducible factor-1α regulates cell proliferation, migration and invasion

Hiroyuki Mori, Yao Yao, Brian S. Learman, Kazuhiko Kurozumi, Joji Ishida, Sadeesh K. Ramakrishnan, Katherine A. Overmyer, Xiang Xue, William P. Cawthorn, Michael A. Reid, Matthew Taylor, Xiaomin Ning, Yatrik M.Shah, Ormond A. MacDougald

Table of Contents

1) Materials and Methods for Supplemental Figures: pg 2

2) Supplemental Figures

Supplementary Figure 1: pg 4
Supplementary Figure 2: pg 6
Supplementary Figure 3: pg 7
Supplementary Figure 4: pg 8
Supplementary Figure 5: pg 10
Supplementary Figure 6: pg 11

MATERIALS AND METHODS FOR SUPPLEMENTAL FIGURES

Primers for Quantitative RT-PCR

Primer sequences for real-time RT-PCR were: *Wnt5a* sense 5'-AATAACCCTGTTCAGATGTCA-3', antisense 5'-TACTGCATGTGGTCCTGATA-3', *Sfrp5* sense 5'-GAAAGTTGATTGGAGCCCAGAA-3', antisense 5'-GCCCGTCAGGTTGTCTAACTGT-3',

Methylthiazol Tetrazolium (MTT) assay

MTT assay was performed using the commercial kit (MTT Cell Proliferation Assay. Kit, Cayman Chemical) according to the manufacture's procedure as follows.

Generation of Wnt11 knockout cells using CRISPR/cas9 genome editing

The 20 nucleotide guide sequences targeting human or mouse WNT11 were designed using the CRISPR design tool at http://crispr.mit.edu/. The guide RNA (gRNA) encoding DNA were cloned into a bicistronic expression vector (LentiCRISPR v2; Addgene plasmid # 52961) containing human codon–optimized Cas9 and the RNA components[1]. The guiding sequence with 3 nucleotides of protospacer adjacent motif (PAM) are shown below. They target exon 3 of both human and mouse Wnt11 genes. Human Wnt11 guide RNA sequence: 5’-GGCCGACAGCGCATACACGAAGG, mouse Wnt11 guide RNA sequence: 5’-GGCCGACAGGGCATACACGAAGG. As a control, a gRNA sequence targeting CRE recombinase while not human nor mouse genome was designed: 5’-GTAAAAATGCCAGGTCGCGCTGG. The LentiCRISPR vector containing different gRNAs were transfected into HEK293T cells with lentiviral packaging plasmid psPAX2 and envelope

plasmid pMD2. The virus were collected and concentrated, and used to infect MDA-MB-231 cells or 4T1 cells, respectively. 24hrs after infection, cells were selected with puromycin 5 days post infection, the cells were examined for Wnt11 deletion, and used for further experiments.

References

1. Sanjana NE, Shalem O, Zhang F. Improved vectors and genome-wide libraries for CRISPR screening. *Nature methods* **11**, 783-784 (2014).

2. Uysal-Onganer P, Kypta RM. Wnt11 in 2011 - the regulation and function of a non-canonical Wnt. *Acta Physiol (Oxf)* **204**, 52-64 (2012).

3. Pepicelli CV, Kispert A, Rowitch DH, McMahon AP. GDNF induces branching and increased cell proliferation in the ureter of the mouse. *Dev Biol* **192**, 193-198 (1997).

4. Lin Z, Reierstad S, Huang CC, Bulun SE. Novel estrogen receptor-alpha binding sites and estradiol target genes identified by chromatin immunoprecipitation cloning in breast cancer. *Cancer Res* **67**, 5017-5024 (2007).

5. Dwyer MA*, et al.* WNT11 expression is induced by estrogen-related receptor alpha and beta-catenin and acts in an autocrine manner to increase cancer cell migration. *Cancer Res* **70**, 9298-9308 (2010).

6. Ueno S*, et al.* Biphasic role for Wnt/beta-catenin signaling in cardiac specification in zebrafish and embryonic stem cells. *Proceedings of the National Academy of Sciences of the United States of America* **104**, 9685-9690 (2007).

7. Gros J, Serralbo O, Marcelle C. WNT11 acts as a directional cue to organize the elongation of early muscle fibres. *Nature* **457**, 589-593 (2009).

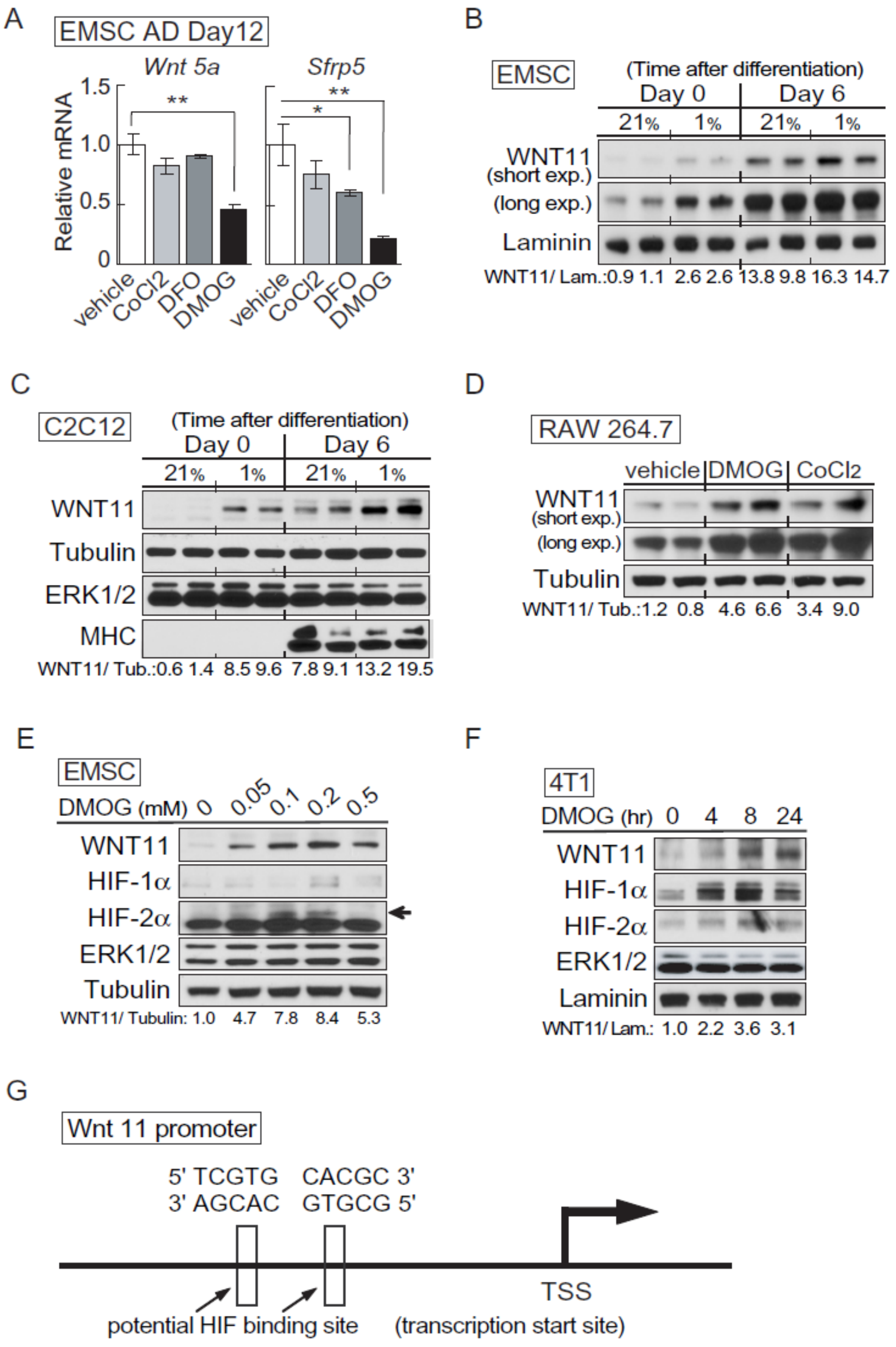
A
EMSC AD Day12
Wnt 5a
Sfrp5
Relative mRNA
1.5
1.0
0.5
0
**
*
**
vehicle
CoCl2
DFO
DMOG
B
EMSC
(Time after differentiation)
Day 0
Day 6
21%
1%
WNT11
(short exp.)
(long exp.)
Laminin
WNT11/ Lam.:0.9 1.1 2.6 2.6 13.8 9.8 16.3 14.7
C
C2C12
(Time after differentiation)
Day 0
Day 6
21%
1%
WNT11
Tubulin
ERK1/2
MHC
WNT11/ Tub.:0.6 1.4 8.5 9.6 7.8 9.1 13.2 19.5
D
RAW 264.7
vehicle
DMOG
CoCl2
WNT11
(short exp.)
(long exp.)
Tubulin
WNT11/ Tub.:1.2 0.8 4.6 6.6 3.4 9.0
E
EMSC
DMOG (mM) 0 0.05 0.1 0.2 0.5
WNT11
HIF-1α
HIF-2α
ERK1/2
Tubulin
WNT11/ Tubulin: 1.0 4.7 7.8 8.4 5.3
F
4T1
DMOG (hr) 0 4 8 24
WNT11
HIF-1α
HIF-2α
ERK1/2
Laminin
WNT11/ Lam.: 1.0 2.2 3.6 3.1
G
Wnt 11 promoter
5' TCGTG CACGC 3'
3' AGCAC GTGCG 5'
potential HIF binding site
TSS
(transcription start site)

Supplemental Figure 1

Wnt11 is induced by hypoxia or hypoxia-mimetic agents in different cell types.

(**A**) Neither *Wnt5a* nor *Sfrp5* was induced by hypoxia mimetics. EMSC adipocytes were treated with $CoCl_2$ (0.1 mM), DFO (0.1 mM) or DMOG (0.1 mM) for 24 hrs same as Figure 1A. Values were normalized to *Tbp* mRNA and are expressed relative to control (*n* = 3). (**B** and **C**) Increased WNT11 protein expression by hypoxia in EMSC preadipocytes and adipocytes (Day 0-12 after differentiation) (**B**), and C2C12 myoblast and myocyte (Day 0 and 8 after differentiation). Myosin heavy chain (MHC) as a differentiation marker (**C**). RAW 264.7 cells after treatment with hypoxia-mimetic agents for 24 hrs (**D**). (**E**) Increased WNT11 by DMOG treatment in a dose-dependent manner. EMSC were treated with indicated dose of DMOG for 24 hr. (**F**) Time course experiment from 4T1 cells treated with 0.1 mM DMOG. (**G**) Diagram of the luciferase reporter constructs of Wnt11. 1064bp DNA fragment in mouse Wnt11 promoter proximal region was cloned into XhoI/HindIII sites on pGL3-basic plasmid. For panels of immunoblotting, laminin, α-tubulin, and ERK were used as loading controls, WNT11 normalized to α-Tubulin was shown.

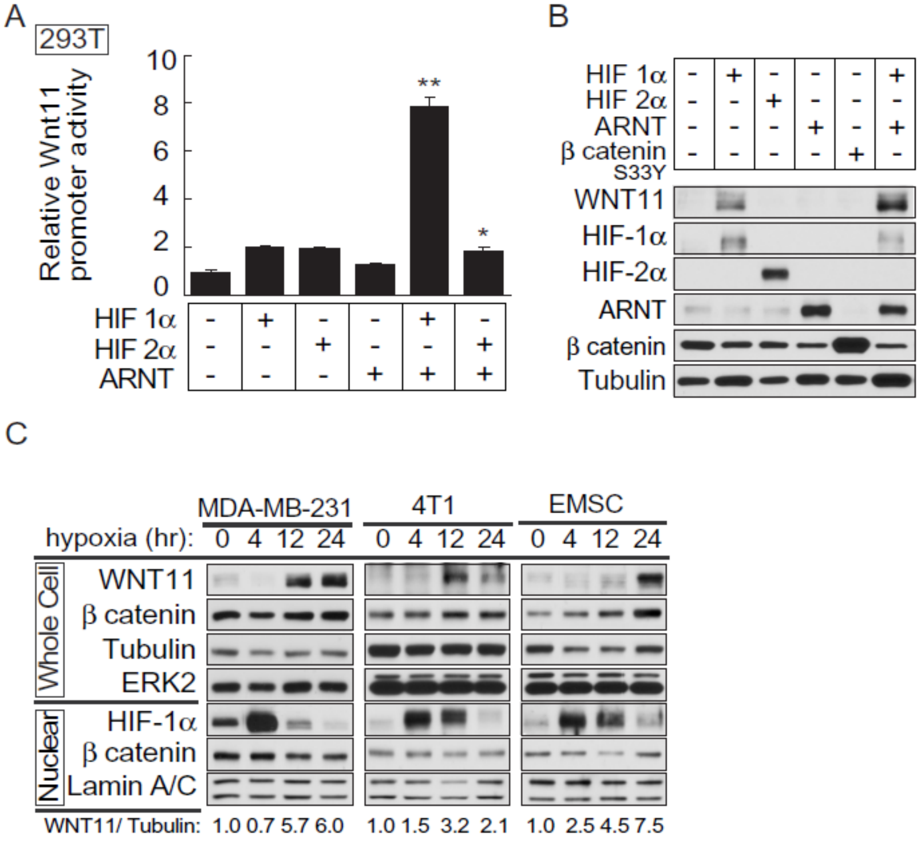

Supplementary Figure 2

(**A**) Co-transfection of Hif-1α and Arnt induces *Wnt11* promoter activity. Constructs encoding Hif-1α, Hif-*2*α or Arnt were transfected as indicated with *Wnt11* promoter-luciferase reporter into HEK293T cells. Luciferase activities were measured 48 hrs after transfection. (**B**) Immunoblotted with indicated antibodies are shown the expression of endogenous and exogenous expression of each protein in HEK293T cells related to luciferase data. (**C**) No further accumulation of nuclear β-catenin under hypoxia. Immunoblots of MDA-MB-231, 4T1 and EMSC incubated under 1% O_2 for the indicated times. ERK, α-tubulin and Lamin A/C were used as loading controls, WNT11 normalized to α-Tubulin was shown.

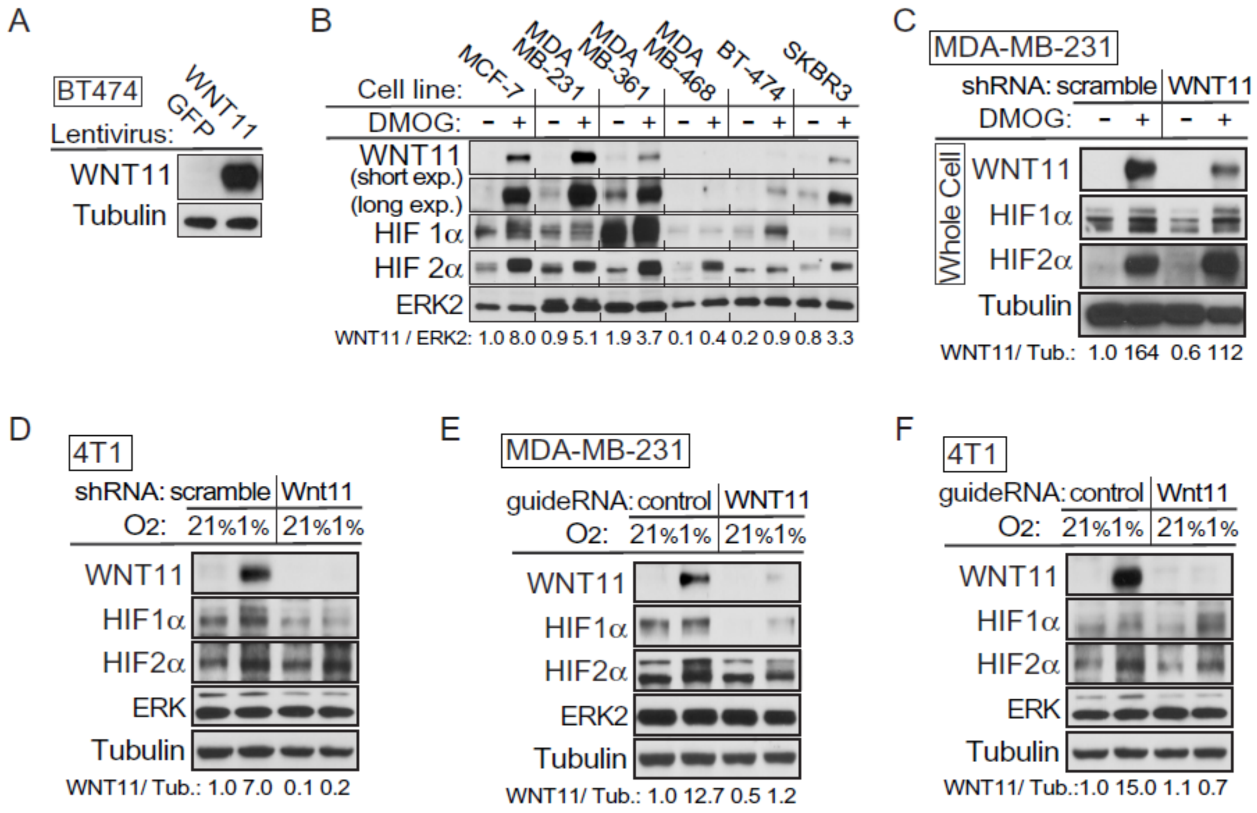

Supplementary Figure 3

(**A**) Expression of lentiviral-derived WNT11 in BT474 cells under normoxic conditions. (**B**) Relative induction of WNT11, HIF-1α and HIF-2α in cancer cell lines with DMOG. (**C**) MDA-MB-231 cells, which have amongst the highest WNT11 expression levels of cell lines tested, stably expressing an shRNA against WNT11 or scrambled control were treated with 0.1 mM DMOG and analyzed by immunoblotting. (**D**) 4T1 cells stably expressing shRNA against Wnt11 or scrambled control were treated under 21% O_2 or 1% O_2. (**E** and **F**) Detection of WNT11 knockout MDA-MB-231 cells (**E**) and 4T1 cells (**F**) by gRNA approach, WNT11 normalized to α-Tubulin was shown.

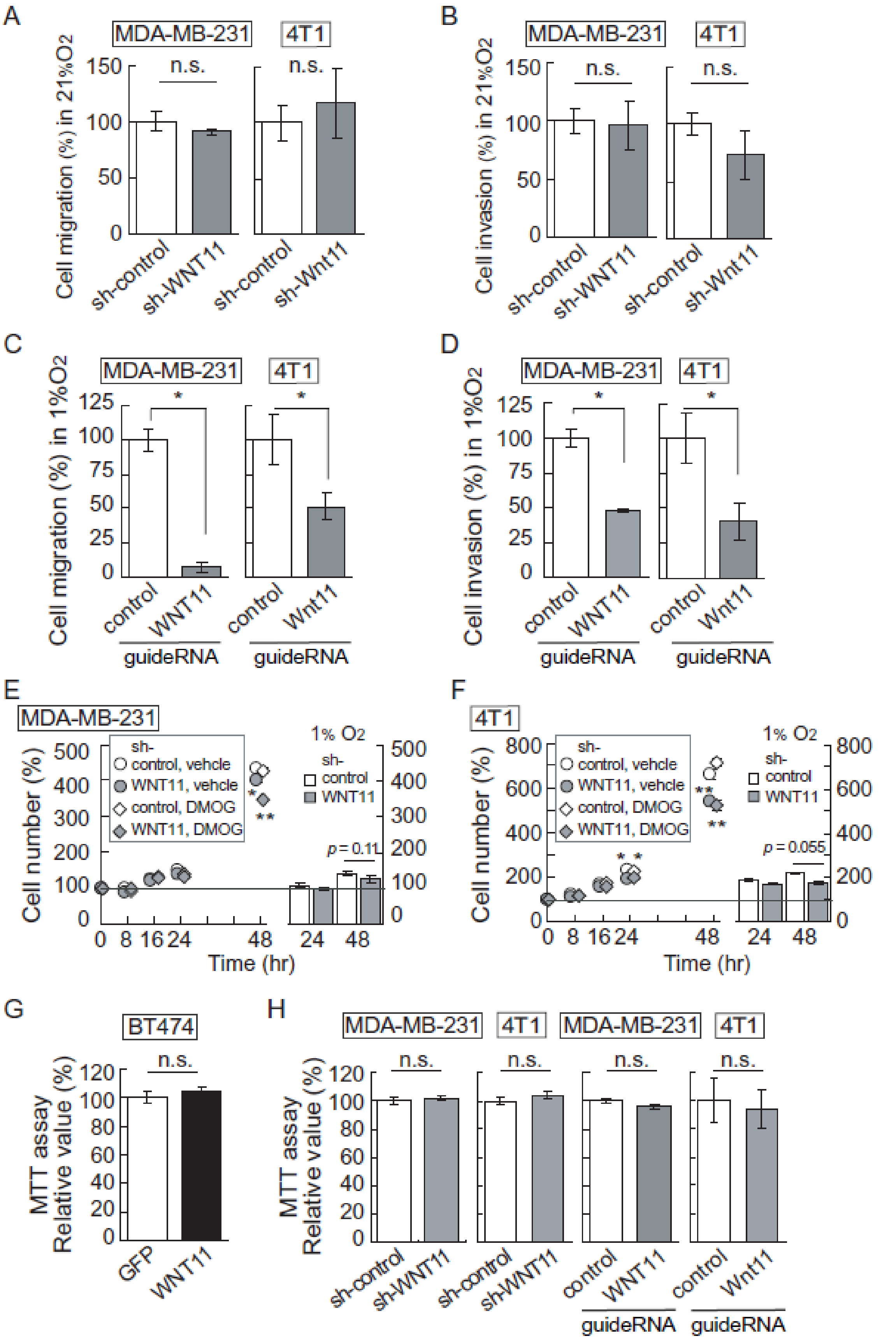

A
MDA-MB-231
4T1
n.s.
n.s.
Cell migration (%) in 21%O2
sh-control
sh-WNT11
sh-control
sh-Wnt11
B
MDA-MB-231
4T1
n.s.
n.s.
Cell invasion (%) in 21%O2
sh-control
sh-WNT11
sh-control
sh-Wnt11
C
MDA-MB-231
4T1
Cell migration (%) in 1%O2
control
WNT11
guideRNA
control
Wnt11
guideRNA
D
MDA-MB-231
4T1
Cell invasion (%) in 1%O2
control
WNT11
guideRNA
control
Wnt11
guideRNA
E
MDA-MB-231
Cell number (%)
sh-
control, vehcle
WNT11, vehcle
control, DMOG
WNT11, DMOG
1% O2
sh-
control
WNT11
p = 0.11
Time (hr)
F
4T1
Cell number (%)
sh-
control, vehcle
WNT11, vehcle
control, DMOG
WNT11, DMOG
1% O2
sh-
control
WNT11
p = 0.055
Time (hr)
G
BT474
n.s.
MTT assay
Relative value (%)
GFP
WNT11
H
MDA-MB-231
4T1
MDA-MB-231
4T1
n.s.
n.s.
n.s.
n.s.
MTT assay
Relative value (%)
sh-control
sh-WNT11
sh-control
sh-WNT11
control
WNT11
guideRNA
control
Wnt11
guideRNA

Supplementary Figure 4

Reduced expression of WNT11 did not influence cell migration (**A**) or invasion (**B**) under normoxic conditions. Boyden chamber assays were used to analyze migration or invasion of MDA-MB-231 and BT-474 cells. WNT11 knockout by CRISPR-Cas9 in MDA-MB-231 cells or 4T1 cells showed impaired migration (**C**) and invasion (**D**) under hypoxic conditions. Cells were incubated with 0.2 mM DMOG for 8 hrs to induce WNT11 expression. Then cells were seeded in the Boyden chamber and incubated under hypoxic conditions (1% O_2) for 16 hr. WNT11 deficiency decreased number of MDA-MB231 (E) and 4T1 (F) cells after incubation for 48 h under normoxic or hypoxic conditions. Cell number was manually counted under a light microscope at indicated times after seeding. No difference in MTT assay in cells stably overexpressing WNT11 (G), or stably expressing shRNAs against WNT11 (H) during migration and invasion assays under hypoxia. For all panels, values are mean ± s.e.m. *p<0.05, **p<0.01.

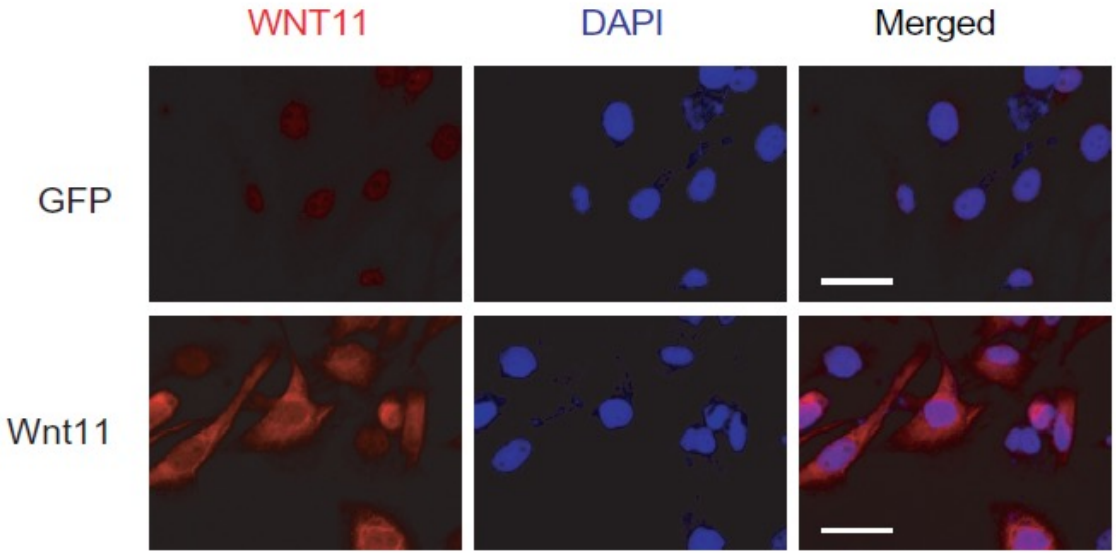

Supplementary Figure 5

WNT11 antibody was validated using overexpression of Wnt11 in MDA-MB-231 cells. Detection of Wnt11 (red), DAPI (blue) and their colocalization (right) are shown. The scale bar represents 50 μm.

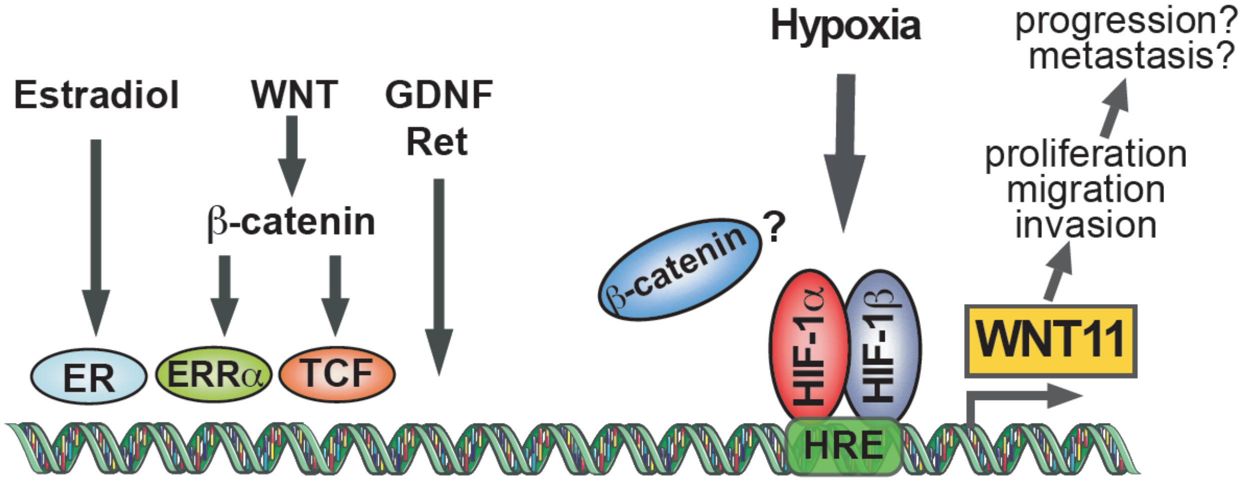

Supplementary Figure 6

Several factors have been reported to regulate Wnt11 expression[2]; Ret/GDNF signaling[3], estrogen (ER) /estrogen-related receptor α (ERRα)[4, 5], β-catenin[5] and TCF/LEFs [2, 4, 6, 7]. Here we report that WNT11 is induced by hypoxia, β-catenin is required for WNT11 expression, and that transcription of WNT11 is regulated primarily by HIF-1α. WNT11 stimulates proliferation, migration and invasion, and increases activity of matrix metalloproteinase (MMP)-2 and 9. We propose that WNT11 as a possible target for cancer therapies. This panel was produced using Servier Medical Art.
